# Hospital personnel perspectives on factors influencing acute care patient outcomes: a qualitative approach to model refinement

**DOI:** 10.1186/s12913-024-11271-x

**Published:** 2024-07-12

**Authors:** Jessica Ziemek, Natalie Hoge, Kyla F. Woodward, Emily Doerfler, Alison Bradywood, Alix Pletcher, Abraham D. Flaxman, Sarah J. Iribarren

**Affiliations:** 1 School of Nursing, University of Washington, Box 357260, Seattle, WA 98195 USA; 2 Washington Board of Nursing, PO Box 47864, Olympia, WA 98504 USA; 3 University of Washington Institute for Health Metrics and Evaluation, Box 351615, Seattle, WA 98195 USA

**Keywords:** Staffing, Workforce, RN staffing, Care models, Qualitative

## Abstract

**Background:**

Health systems have long been interested in the best practices for staffing in the acute care setting. Studies on staffing often focus on registered nurses and nurse-to-patient staffing ratios. There were fewer studies on the relationship between interprofessional team members or contextual factors such as hospital and community characteristics and patient outcomes. This qualitative study aimed to refine an explanatory model by soliciting hospital personnel feedback on staffing and patient outcomes.

**Methods:**

We conducted a qualitative study using semi-structured interviews and thematic analysis to understand hospital personnel’s perspectives and experiences of factors that affect acute care inpatient outcomes. Interviews were conducted in 2022 with 38 hospital personnel representing 19 hospitals across Washington state in the United States of America.

**Results:**

Findings support a model of characteristics impacting patient outcomes to include the complex and interconnected relationships between community, hospital, patient, and staffing characteristics. Within the model, patient characteristics were positioned into hospital characteristics, and in turn these were positioned within community characteristics to highlight the importance of setting and context when evaluating outcomes. Together, these factors influenced both staff characteristics and patient outcomes, but these two categories also share a direct relationship.

**Conclusion:**

Findings can be applied to hospitals and health systems in a variety of contexts to examine how external factors such as community resource availability impact care delivery. Future research should expand on this work with specific attention to how staffing changes and interprofessional team composition can improve patient outcomes.

**Supplementary Information:**

The online version contains supplementary material available at 10.1186/s12913-024-11271-x.

## Introduction

Acute care health systems face ongoing challenges in recruiting and retaining staff to meet the needs of their patients. Best practices in acute care staffing have long been a topic of interest for organizations around the world [1]. As demonstrated in a recent systematic review covering two decades of research, studies often focus exclusively on the impact of registered nurse (RN) staffing on patient outcomes [1]. However, patient care is also impacted by staffing levels of other clinical and nonclinical care team members [2], and outcomes are also influenced by organizational and community factors (termed *contextual factors)* [3–5]. For example, in a community with limited skilled nursing facility beds, patients needing this level of care after discharge may experience longer hospital stays [6], exposing them to risks from adverse events such as inpatient falls or hospital-acquired infections. A better understanding of care team staffing, contextual factors, and their impacts on patient outcomes is vital to ensuring the development and implementation of meaningful policy related to healthcare staffing.

In 2021, the Washington (WA) state legislature passed a bill focused on transparency in healthcare [7]. The bill directed the state Department of Health to commission an interdisciplinary team to engage hospital personnel throughout the state and examine the relationships between the acute care workforce and patient outcomes by systematically investigating how workforce characteristics such as the number, type, education, training, and experience of staff affects patient mortality and other patient outcomes [7]. Our team, led by the University of Washington (UW) School of Nursing in collaboration with researchers at the UW Institute for Health Metrics and Evaluation, was selected to conduct this study. To carry out this research, we established partnerships with contributors including hospital leaders, healthcare associations, and union representatives, to ensure that we addressed the multiple contextual elements impacting patient care outcomes as well as examining staffing of the care team more inclusively. The project included four phases: 1) reviewing studies which examined the impact of care team characteristics such as experience and education on patient outcomes; 2) developing a preliminary explanatory model and analysis plan based on the review, contributor input, and available data sources; 3) refining the model using qualitative data from hospital personnel; and 4) completing a quantitative analysis utilizing anonymized state- and hospital-collected healthcare data guided by the refined model.

**Fig. 1:**
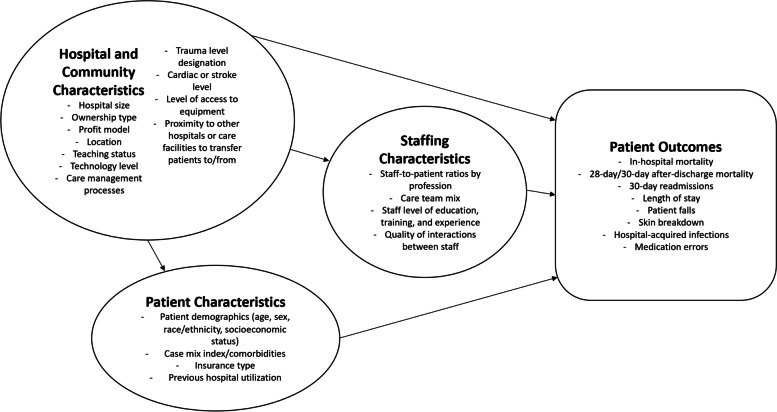
Initial model of factors impacting staffing and patient outcomes in acute care hospitals. This model drew on findings from the initial phases of the study including literature review and feedback from key contributors across the state [8]

In phase one, we completed a review of systematic reviews that identified several gaps in existing data [8]. First, outside of RN staffing ratios or nursing team composition ratios (e.g., RNs and nursing assistants), staffing of the expanded acute care team, including professionals such as therapists or social workers and nonclinical workers such as environmental services, was rarely quantified in health services literature linking staffing to patient outcomes. The second gap was the limited inclusion or assessment of hospital- and community-level factors in studies examining the relationship between staffing and patient outcomes [8]. The lack of defined factors and the absence of clear frameworks led us to co-develop an explanatory model with key contributors in phase two (Fig. 1) [8]. The purpose of phase three was to assess and refine the model with input from hospital personnel, including identification of missing factors and how all the factors may have contributed to outcomes in various acute care contexts, including impacts on operations, work environment, quality, and outcomes for patients and workers. This paper reports on the process of iterative model refinement by 1) presenting the perspectives of hospital personnel across WA on the preliminary model and factors, and 2) showing how these findings were integrated to refine the model. In the final phase of the project, factors identified in the present study were operationalized for use as independent and control variables in our quantitative analysis of the relationship between staffing and patient outcomes in the state [9].

## Methods

### Study design

We conducted a qualitative study using semi-structured interviews and thematic analysis to understand hospital personnel perspectives and their experiences of factors that affect acute care patients’ outcomes. To refine our model, qualitative research methods were chosen to explore relationships between factors, including context, mechanisms, and outcomes [10]. This exploratory approach acknowledges the significance of subjectivity in the data and allows for inductive inquiry [11]. As a critical component of modeling, an iterative process included updating the model following multiple interviews and then further assessing and revising with subsequent participants. The findings from this study served as a foundation for developing a comprehensive model we called the ‘WA Acute Care hospital Characteristics and patient Outcomes model’ (WACCHO), which considers community, hospital, and patient characteristics that interact with staffing to affect patient outcomes. This study was granted exempt status by the UW and the WA Institutional Review Boards (STUDY00013975).

### Participant recruitment

Participants were purposively sampled through announcements targeting acute care hospital administrators and representatives sent to state-wide open subscription hospital email listservs managed by the WA Department of Health. Hospital executives and administrators were also directly emailed to increase participation and ensure participants represented critical access and general acute care hospitals in various regional settings as designated by WA state. Site contacts were asked to invite any hospital representative(s) who could provide perceptions of staffing’s impact on patient outcomes to the interview, so interviews frequently included several participants. Participants were unknown to the research team prior to the interviews.

### Data collection

The preliminary explanatory model from study phase 2 was used to develop a semi-structured interview guide which was shared with participants prior to the interview. The model was used to guide exploration of agreement, disagreement, and identification of missing factors from each category of the model. Model categories included hospital characteristics and external factors, patient characteristics, staffing characteristics, and patient outcomes. Open-ended questions explored factors, mechanisms, contextual elements, and additional factors that could potentially influence patient outcomes. Interviewers also presented facility-specific data, asked participants for their perceptions of accuracy, and discussed the basic analysis plan for the quantitative portion of the study. A list of interview questions and prompts is provided in Additional File 1. Interviews were conducted between January and June 2022 via video conferencing by two to five members of the research team. Each participant was interviewed once, either individually or with other participants from the same organization, and all interviews were audio recorded and transcribed for analysis. The research team introduced themselves and explained the purpose of the study. Upon obtaining oral consent from participants, the team conducted the interview, and two members of the research team (SI, NH) took detailed field notes. Transcripts were uploaded to ATLAS.ti (version 9).

### Analysis

Both deductive and inductive methods were used in the thematic analysis of data [11]. An initial codebook was created based on our initial model and interview notes, and emergent codes were added inductively during analysis [5]. Five team members (NH, JZ, ED, KN, KB) contributed to coding. They met weekly to review codes, ensure a uniform interpretation and application of the coding framework, and address any discrepancies. At least two researchers coded a portion of each transcript to ensure consistency. Once coding was completed, codes were iteratively organized into main themes and subthemes to capture the range of narratives [5]. Data saturation was determined when no new themes were identified in final interviews [12]. We followed the consolidated criteria for reporting qualitative research guidelines (COREQ) to ensure comprehensive reporting [13].

## Results

### Participants

A total of 20 interviews were conducted with 38 participants from 19 hospitals in eight out of nine regions across WA. Participants worked at three main types of hospitals: acute care (23/38, 60%), critical access (11/38, 29%), and sole community hospitals (4/38, 11%). While the definitions of hospital types may vary in some literature, the Centers for Medicare and Medicaid Services (CMS) officially designates critical access and sole community hospitals as specific types of acute care hospitals which are typically smaller and located in rural settings [14, 15]. Participants included a broad range of executives and administrators (23/38, 61%), directors and managers (10/38, 26%), and care team members (5/38, 13%) from all three hospital types. Mean interview length was 61 min.

### Explanatory model

**Fig. 2:**
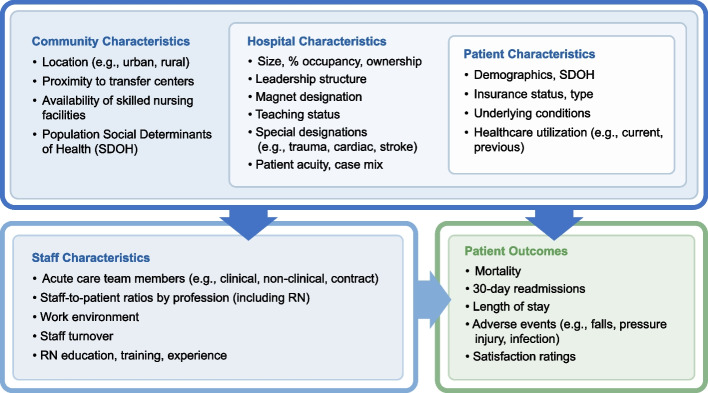
Refined explanatory model of factors impacting staffing and patient outcomes in acute care hospitals. Study findings were used to refine and enhance the initial model, developing an explanatory model for use in subsequent phases of the project

The final explanatory model represents the primary common factors and drivers impacting patient outcomes in acute care hospitals based on hospital personnel perspectives (Fig. 2). Changes to the initial model (Fig. 1) [8] included the division of the *external factors* category into *community characteristics* and *hospital characteristics*, positioning of *patient characteristics* into *hospital characteristics* and *hospital characteristics* into *community characteristics* to highlight the interrelatedness between the categories as identified by hospital personnel. The new community characteristics category impacts both staffing characteristics and patient outcomes, while staffing and patient outcomes continue to be directly connected. The following section presents findings organized by model category, and Table 1 provides the number of hospitals out of 19 reporting on each of the main themes within the four model categories.

**Table 1:** Main study themes and frequency by model section

	**Theme**	**Frequency (*n* = 19)**
***Community Characteristics***	Location and community resources	13
	Population characteristics	15
***Hospital Characteristics***	Hospital type and access to resources	19
	Hospital leadership structure and culture as foundational to quality	19
	Influence of organizational culture on work environment and staff retention	15
	Units vary across and within hospitals	16
***Patient Characteristics***	Underlying health conditions impact the intensity of care	16
	Social history and economic characteristics impact health status	15
***Staffing Characteristics***	Care team composition and the central role of nurses	17
	Influence of staffing type on work environment	8
	Nurse absorption of non-nursing duties resulting in the dilution of nursing care roles	18
	Education, training, experience	18
***Patient Outcomes***	Impact of staffing on patient outcomes	12
	Cumulative impacts on patient outcomes	11

Frequency represents the number of hospitals whose participants identified factors in each theme

### Community characteristics

Community characteristics were defined as factors outside of the hospital’s control which impacted staffing or patient care, including sociopolitical, geographical, and economic factors, and availability of healthcare resources.

#### Location and community resources

Participants often described the difficulty of discharging patients to the appropriate level of care due to resource and facility availability, including higher acuity transfers to a referral hospital, or discharge to subacute care such as a skilled nursing facility. When resources for the appropriate level of care were limited, the hospital must keep patients in acute care beds, limiting available resources for other patients. These challenges were more pronounced in rural settings with fewer community resources.

Community characteristics also impacted staffing. Both rural and urban participants described how location and community resources like affordable housing, public transportation, and commute times made it difficult to recruit and retain hospital staff. A participant from a metropolitan area hospital noted that,* “Our entry level and mid-level workers cannot afford to work at [hospital name]. They are driving 35, 45 min, an hour, each way just to come to work here.”* Similarly, a chief nursing officer from a hospital in a rural setting also noted housing and commute as community factors impacting staffing, *“Even if I hire somebody and if they're willing to move here, they can't get housing… one of the OR [operating room] nurses I'm losing, is because the commute is too long. It’s about an hour and half for her.”* Additionally, participants in rural locations noted the difficulty in recruiting staff when they were not close to or connected with teaching institutions producing new graduates.

#### Population

Participants discussed their facilities’ unique challenges due to both the populations they served and health disparities present within the community. One acute care hospital administrator listed some of the challenges faced by the populations served by their hospital such as, *“access to core necessities. So, transportation, food, medication, housing...”* Another community level challenge was changing seasonal populations. One hospital administrator at a critical access hospital noted the following: “*One of the things... about out here, is the 300 days of sunshine. We get all kinds of visitors. You know our town is 5000 people, but in the summertime, it would be 30,000 people. They come in for fishing, and the weather, and rodeos and other things. So, I know other critical access hospitals that are in rural areas with water and different things that have faced the same kind of thing. You never know how many people that are going to come in.”* Multiple population characteristics were identified by participants as leading to unique needs in the acute care setting, including homelessness, homes without basic utilities (e.g., running water or electricity), health insurance in the community, and transient seasonal populations including migrant workers and summer tourists.

### Hospital characteristics

Hospital characteristics were defined as the structural and functional qualities of acute care hospitals that influenced the services they offered and the complexity of patients they served.

#### Hospital type and access to resources

Participants stated that their hospital type, specifically size and connection to larger health systems, influenced their access to resources. Critical access hospitals described more limited access to the relationships and knowledge larger health systems share, including inefficiencies in changing practices. As one administrator said,*“… being part of a system really changes things as well, because if it’s a system, then the system itself collaborates and has greater resources to roll forward processes that have been vetted at a higher level.”* Various participants described limited budgets and reduced access to equipment secondary to supply chain constraints. Critical access hospitals identified their smaller size and limited resource pool as reasons they must be more particular with capital investments that would enable them to care for more complex patients while simultaneously having the obligation to provide specialty services that were not otherwise available in their communities.

#### Hospital leadership structure and culture as foundational to quality

Participants considered staffing and leadership culture as a product of organizational priorities that influenced staff satisfaction and quality outcomes. They identified such as access to adequate equipment and supplies as important to providing quality patient care. As one acute care hospital administrator described, “*Something as simple as an overbed table… When we talked about this at incident command…the answer was no. And then, thank God, our CEO is also a nurse and she’s like no, this is basic to taking care of patients and keeping them from falling.”* Participants also listed other structural and cultural characteristics including union status, staffing strategies, and budgets as impacting patient care and patient care outcomes.

Participants noted organizational features that emphasized safety culture, with elements like care quality and improved organizational processes. Multiple participants referenced standardized protocols as a safety tool that contributed to improved patient care. Participants also felt an organizational focus on safety and transparency improved staff satisfaction and quality of care. Comments referenced the importance of continuous quality improvement and a focus on process improvement instead of individual errors.

#### Influence of organizational culture on work environment and staff retention

Participants agreed that the culture of an organization influenced both the work environment and staff retention. They described approaches to support and engage with staff which promoted a positive organizational culture. One approach included providing staff with incentives and benefits such as increased pay, bonuses, parking passes, flexible shifts, and scheduling. Other examples included programs which covered the cost of nursing education in exchange for commitment to work in a given facility for a period of time. Consequently, insufficient organizational culture can lead to staff turnover, as noted by one care team member*, “if you’re not given the tools to do your job well, anybody with any empathy is going to go find something else to do.”* Participants also presented upstream approaches which improved the work environment, such as involving workers in organizational decision making and appropriate staffing of the interprofessional team.

#### Units vary across and within hospitals

When discussing data metrics, participants often discussed the difficulty in making comparisons of the same unit between different hospitals and comparisons of units within the same hospital. They expressed confusion with how acuity is defined, especially when comparing patient care across different facilities. Participants felt it was too difficult to use case mix index, a metric used to identify the diversity and severity of patients cared for at specific hospitals, to compare outcomes between units within a hospital or across healthcare systems. Participants did not think case mix encompasses all the variables that should be considered when evaluating the complexities of the patient and the care infrastructure.

### Patient characteristics

Patient characteristics were defined as individual demographic, social, and health characteristics of patients admitted to the hospital that may impact the level of care needed.

#### Underlying health conditions impact the intensity of care

Participants used the term ‘care intensity’ to describe how patient care needs impacted work demands on staff, with agreement that the care intensity is not always directly tied to the patient’s admitting diagnoses or assigned acuity. Participants reported this disparity between acuity and care intensity as a challenge to accurately predict staffing needs. They noted that specific health conditions with higher care intensity included aggressive behavior, traumatic brain injury, obesity, substance use, and dementia. When discussing resource intensive patients, one participant described that, *“it generally is a lot of, uh, psychosocial intervention for these people... it’s usually not necessarily relatable to what the acuity of their medical diagnosis is. In fact, it’s frequently not. So it almost needs to be on its own scale, acuity scale... to really accurately reflect the amount of staff time it takes.”*

Participants described different strategies to account for care intensity variations, such as having a centralized staffing office or a predefined team who coordinated activities to accommodate rapid and fluctuating changes in staffing needs. In addition to increased care intensity and inpatient staffing demands, patients with certain underlying conditions were difficult to discharge due to the availability of appropriate care in the community or mandated social supports such as individuals needing guardian assignment.

#### Social history and economic characteristics impact health status

In addition to overall population characteristics, individual demographics, social determinants of health (SDOH), and insurance status of patients influenced their care needs. Factors such as access to routine care, prior healthcare utilization, and comorbidities impacted care intensity and resources needed for patient care.

### Staffing characteristics

Staffing characteristics were defined as acute care team members, their roles, and aspects of staffing which influence how facilities provide staff and deliver patient care.

#### Care team composition and the central role of nurses

When considering the relationship between staffing and patient outcomes, participants discussed team members who contribute to the care team and work in tandem to provide patient care. Participants mentioned roles in multiple professions including physicians, advanced practice providers, RNs, certified nursing assistants, occupational therapists, PTs, pharmacists, social workers, dietary aides, environmental service workers, billing/coding staff, students, and others. Care team members were generally categorized as either clinical, non-clinical, or temporary roles. There was a lack of agreement around the types and breadth of roles included in the acute care team. However, participants discussed state mandated annual RN staffing plans and nurse-to-patient ratios, highlighting the significant role and value placed on RNs in acute patient care and care teams.

#### Influence of staffing type on work environment

Participants emphasized the importance of differentiating between temporary (e.g., contract, agency, or travel) and permanent RNs when examining how staffing impacted patient outcomes. Participants expressed that temporary workers may be less familiar with facility policies and may not have the same unit-specific training as permanent staff. Additionally, facilities with a larger proportion of rotating temporary workers may not have an established culture of communication and support, which diminishes the quality of the work environment and negatively influences patient outcomes.

#### RN absorption of non-nursing duties resulting in the dilution of nursing care roles

Participants presented instances when facilities had difficulty filling staffing roles, so RNs absorbed responsibilities, diluting the scope of nursing practice. For example, one sole community hospital administrator stated, *“If you’re short PT assistants or PT aids, that falls back on the RN and the nursing assistant. If you don’t have case management or social work, that also falls on the RN. Everything falls on the RN, if... the rest of the team is missing.”* Although facilities submit annual nurse staffing matrices, participants frequently spoke to the need to deviate from planned models, highlighting variation in direct and indirect patient support staff which make nurse-to-patient ratios in one setting incomparable to the same workload in another setting.

#### Education, training, and experience

Discussions around education, training, and experience centralized around nursing staff and focused on the nuances of the term ‘experience.’ Participants agreed that RN experience was complex and difficult to capture, quantify and standardize. Various metrics for measuring experience were presented and considered, such as years of RN or inpatient experience and unit tenure. Participants also quantified RN experience with standards such as a novice to expert or years since licensure. Degrees, licenses, and certifications were discussed as components of education, with several participants stating that RN training was not well documented except in human resource records. Participants noted that overall training and experience on the unit influenced their ability to staff appropriately for patient acuity and diagnosis. When units had higher numbers of staff with more training and experience, the unit could manage more complex patients, yet in many locations, the limited number of experienced staff made patient assignments difficult.

### Patient outcomes

Patient outcomes included metrics pertaining to characteristics of a patient’s stay at a hospital and the time immediately following discharge, which were a collection of quality and safety metrics tracked by the hospital and the state.

#### Impact of staffing on patient outcomes

When asked about patient outcomes, participants described some measures as more sensitive to staffing than others. Participants specifically mentioned falls and pressure ulcers as staffing-sensitive outcomes, with one hospital administrator noting that, *“one of the things…making a significant impact on patient outcomes or patient satisfaction and staffing is the number of non-hospital nurses that we have here. So, we have 72 travelers, and we have FEMA [Federal Emergency Management Agency] staff, and so our fall rates increased, our HAPIs [hospital-acquired pressure injuries] have increased, complaints have increased.”* Participants characterized staffing-susceptible outcomes as being dependent on care team composition and staffing type rather than the specific number of staff or staff-to-patient ratios.

#### Cumulative impacts on patient outcomes

Several participants described the influence of community and patient characteristics on patient outcome metrics. An example of this is length-of-stay, defined as the number of days a patient is cared for in an acute care facility. One critical access hospital administrator stated, *“it happens, where we cannot get a patient out. We don’t have a receiving hospital or we don’t have EMS [Emergency Medical Services]... that’s the challenge of being... rural.”* Length of stay and other outcomes like readmission rates were also significantly impacted by factors outside of staffing control, for example when patients need social support or skilled nursing care that is not readily available in the community at the time of discharge.

## Discussion

This study produced critical findings on factors influencing staffing and patient outcomes in the acute care setting. Some findings reinforce existing knowledge–such as the importance of adequate RN staffing–and others confirm gaps in both knowledge and theory related to care team staffing more broadly and strategies to account for different resource availability in diverse settings. The discussion highlights the gaps in each of the categories in our model with the knowledge that factors are often interconnected and responsive to dynamic changes in other model components. For example, changes in hospital leadership may impact both hospital and staffing characteristics in ways that subsequently change patient outcomes, and changes in community infrastructure or policy can impact access to health resources.

### Community characteristics

Participants practiced in a wide array of settings and consistently brought forward the need to account for different contexts when considering healthcare staffing policy. Findings suggest that a ‘one size fits all’ approach to staffing is undesirable, instead emphasizing the need for individual organizations to account for their communities and settings when establishing staffing standards and setting outcome targets [16]. This viewpoint is consistent with implementation science theories such as the Consolidated Framework for Implementation Research [17], which emphasizes the inclusion of contextual factors when planning, developing, implementing, and evaluating a practice or policy change. Accounting for community contexts allows organizations to attend to the populations they serve and the resources available in their settings. For example, communities with lower demand for inpatient beds and more limited access to skilled nursing facilities may need the flexibility to provide a lower level of care (e.g., a higher patient to nurse ratio) when a patient ready for skilled nursing is still physically present in the hospital [6].

### Hospital characteristics

While organizational culture has been linked with workforce outcomes such as RN turnover and retention [18], participants indicated that elements of culture were also vital to conversations about staffing and patient outcomes. An organizational emphasis on safety and just culture provides opportunities for workers to provide input on staffing needs and challenges. Within just culture, transparency and psychological safety work bidirectionally to ensure that staff can bring forward concerns without penalty and that management and administration share information on their own challenges and progress related to staffing [19].

Several proven strategies for approaching this type of culture are Magnet® designation, which emphasizes the involvement of RNs in hospital administration, policy, and practice [20], and American Association of Critical Care Nurses’ Healthy Work Environment, which identifies 6 critical elements to a just unit culture [21]. Accounting for features of organizational culture using an established framework such as these would help provide additional information and clarity into organizational practices around staffing, which may be an important predictor or mediator of the relationship between staffing and patient outcomes.

Hospital environment and culture impact patient care in other ways. For example, one participant’s recollection of a discussion about bedside tables shows how a leader with bedside experience recognized the importance of a piece of equipment in promoting patient safety. In addition to these administrative types of decisions, structural and logistical features of hospitals impact staffing and workload. For example, when patient care supplies were not readily available, nurses or other direct care staff had to leave the unit to retrieve them, taking time and focus away from patient care.

### Patient characteristics

Patients with different types of acute care issues had various levels of need, often represented in terms of patient acuity or some measure of nursing hours invested in care [8]. In our study, despite consensus across participants that the unique care needs of individual patients were not routinely captured in acuity measures or admitting diagnoses, there was no agreement on a standardized way these needs could be measured or reported. High care intensity, stemming from the intersection of behavioral, mental, and physical health status, required additional work from the care team. Participants indicated that these situations disrupt the unit's workflow and change staffing needs, even when no additional staff were available. While care quality initiatives aim to increase inpatient assessment of SDOH, these assessments may indicate a need for more resources than staff have available to address issues. Overall, a more nuanced understanding of patient care intensity as it affects the unit workload is necessary when evaluating staffing practice and policies.

### Staffing characteristics

Staffing has been a topic of interest in health services literature for decades, with most data focused on RN staffing levels [1]. One of the main issues identified in our scoping review and reiterated by participants in this study was the lack of consistency around defining a ‘care team’, with terminology like *interprofessional* or *multidisciplinary *teams excluding nonclinical team members and the relative absence of any non-nursing roles in staffing plans or evaluation [8]. Existing data show the essential nature of interprofessional teams in optimizing patient outcomes [22, 23], but focus almost exclusively on teams with clinical roles rather than supportive roles and services. In this study, participants brought forward concerns about what work the RN is doing when other staff were missing and how doing that work impacted their availability to perform needed nursing tasks. Diluting RN time with non-nursing tasks means that RNs were not working at the top of their scope of practice, which leads to dissatisfaction and connects to burnout and turnover [24]. Similarly, when there were not enough RNs with the training and experience to care for certain types of patients, patient outcomes may suffer [25]. In order to develop meaningful policy related to staffing, a more inclusive and holistic definition of the care team is required.

### Patient outcomes

When assessing patient outcomes in health services research, data are often sourced from statewide administrative bodies and include a range of quality metrics such as falls, skin breakdown, length of stay, mortality, and patient satisfaction. While measures like falls and skin breakdown are often labeled as “nursing sensitive”, participants indicated that nurses were not the only staff members whose presence or tasks may impact those outcomes. For example, if typical resources such as PT or PT aides were unavailable to ambulate a patient, the RN may not be able to add that task to their workload, leading to skin breakdown. In this case, the ‘nurse-sensitive’ indicator may not tell the whole story about staffing.

Other patient outcomes like length of stay or readmission may be more indicative of community resources. For example, the availability or staffing levels of residential facilities that care for patients with sequelae of brain injury may mean that patients linger in the acute care setting or get sent back to the emergency room if facility staff were unable to handle symptoms. These types of influences are rarely accounted for in studies which focus on direct measures of nursing staffing and patient outcomes in acute care.

Patient outcomes also vary when underlying conditions or characteristics, including SDOH, impact overall health and complexity of services needed in the acute care setting [26]. WA state now requires hospitals to report certain data on SDOH to the Department of Health [7], which will improve the ability to account for these characteristics in future analyses of patient outcomes and provide more conclusive evidence related to health equity in different patients and communities.

### Implications

Altogether, our findings provide a framework for examining relationships between staffing and patient outcomes more robustly, including components which are currently missing in most health services and health workforce research. Our refined model can be used to guide examination of the ‘new normal’ experienced in healthcare settings following the pandemic, where staffing of multiple care team roles has been unstable and community and organizational characteristics may undergo substantial change.

Findings also reinforce the difficulty of applying a blanket nurse staffing policy to individual organizations. To ensure safe staffing levels at the local, state, or national level, policy needs to reflect more than just the numbers of a specific type of staff at the bedside, instead drawing on a more comprehensive understanding of the communities, settings, and patients served at different facilities. This process may require more robust data collection and policy and budget commitment to ensuring an adequate supply of healthcare workers to achieve high quality outcomes.

### Limitations

This study had several notable limitations. First, the timing of interviews during the COVID-19 pandemic made it challenging for hospital representatives to participate. Frontline staff were often unavailable, and leaders were frequently supporting patient care activities during surges in admissions. This resulted in less robust representation from some care team members and more prominent representation of executive and administrator perspectives, which may have focused the discussion on nurse staffing structures rather than perceptions of patient needs. Second, as the study focused on experiences before the pandemic, participants were asked to remember past perceptions, which challenged their focus and could have led to limited recall bias. Finally, as our model was iteratively developed throughout the interview period, interview questions were not static and discussion may have focused on elements that participants felt more strongly about, influencing the quantity of participant feedback on specific elements of the model.

## Conclusion

Altogether, this study enhanced the initial findings of our scoping review by providing insight from healthcare personnel in several types of acute care hospitals across the state. Findings highlight the complexity and interrelatedness of the categories in the model, while drawing attention to critical gaps that must be addressed to better understand how communities, organizations, patients, and staffing all impact patient outcomes. Our study highlights the need to ensure that RN-centered care teams include appropriate care team staffing to meet the needs of patients, and that access to community resources is critical both for ensuring that patients receive efficient continuity of care throughout their recovery and seeing that acute care beds and staff are appropriately used. Future research should expand on this study to better understand lessons learned from the COVID-19 pandemic and the ‘new normal’ state of healthcare, with specific attention to staffing changes and care team composition that can direct future work to improve patient outcomes. Ensuring optimal staffing of care teams also has the potential to decrease burnout, leading to improved outcomes for acute care staff and improved retention of this vital workforce.

### Supplementary Information

Additional file 1. Semi-structured interview guide. Guide includes a list of the questions and prompts used in stakeholder interviews.

## Data Availability

The participants of this study did not give written consent for their data to be shared publicly, so due to the sensitive nature of the research supporting data is not available.

## Abbreviations

RN: Registered nurse
WACCHO: Washington Acute Care hospital Characteristics and patient Outcomes model
CMS: Centers for Medicare and Medicaid Services
CEO: Chief executive officer
PT: Physical therapist
FEMA: Federal Emergency Management Agency
HAPI: Hospital-acquired pressure injury
SDOH: Social determinants of health
