# Supplementary material for: Hospital personnel perspectives on factors influencing acute care patient outcomes: a qualitative approach to model refinement
Source: BMC Health Serv Res. 2024 Jul 12;24:805. doi: 10.1186/s12913-024-11271-x (PMC11241948; doi:10.1186/s12913-024-11271-x)
Supplement: Supplementary file 1 — Additional file 1. Semi-structured interview guide. Guide includes a list of the questions and prompts used in stakeholder interviews. [file 12913_2024_11271_MOESM1_ESM.docx]

**Additional File 1 (.docx)**

**Semi-Structured Interview Guide**

**Purpose:** The purpose of this study is to assess the relationship between workforce characteristics and patient outcomes (e.g., patient mortality) in acute care hospitals across the State of Washington as directed by Washington State House Bill 1272. *Our goals in this interview are to 1) review existing data sources and 2) get your input on the relationship between hospital staffing and patient outcome as well as the additional factors that might influence this relationship.*

**Existing Data**: We would like to start by reviewing preliminary data from open source data on workforce, patient outcomes, and hospital characteristics of your hospital. XX will present these findings and ask a few questions.

- What are thoughts on this initial analysis?
- If something is identified as incorrect - probe.

**Initial Patient Outcomes Causal Diagram:** Now we would like to get your thoughts on the causal diagram that includes factors that could impact patient outcomes (e.g., 30-day mortality; length of stay; 30-day readmission). We developed this diagram based on the literature and input from key stakeholders. We will review the model from left to right, starting with hospital characteristics. As you are responding to questions, please be sure to specify if you are speaking directly to a specific hospital or network of hospitals. Please note that we will not use names of organizations/hospitals in our report.  Our analysis will make use of hospital-level data, but we will report general relationships and not single out any organization.

- Hospital Characteristics & External Factors
  - Changes/missing & additions
  - Factors of highest importance
  - We are being asked to include the category of **equipment** in our analysis.
    - What types of equipment influence staffing needs or patient health outcomes?
    - Do you have data to capture this information?
    - Who would we talk to to get this information?
  - What **care management** processes are in place at your hospital?
    - Do you have data to capture this information?
- Patient Characteristics
  - Changes/missing & additions
  - Factors of highest importance
  - How is **case mix** categorized or applied to staffing considerations?
    - Do you have data to capture this information?
    - Who would we talk to to get this information?
  - How is patient acuity tracked?
- Staffing
  - Changes/missing & additions
  - Factors of highest importance
  - Who makes up the acute care team? (consider hospital departments/units and hospital staff position)
  - How do you capture staffing data (e.g. FTE/discharge, adjusted discharge with case mix, total hours per adjusted patient day)?
    - At what level of data is staffing captured (unit, hospital)?
    - What is the frequency of the data captured (shift, day, weekly, monthly)?
    - What is the name of the system(s) you use to capture staffing data?
  - Do you have data to capture **education, training, experience** (i.e. NDNQI data or similar data for nursing and other hospital staff)?
  - We need feedback on a list of 81 units/and or occupation categories listed in end of year reports. Would you be willing to review this list and offer feedback? Who might we contact about doing so?
- Patient Outcomes
  - Changes/missing & additions
  - Factors of highest importance
  - Do you have data to capture patient outcomes?
    - At what level (e.g., unit) is the data captured?
    - Is there a variable in the patient outcome dataset which identifies the hospital unit where the patient outcome occurred?
    - Is there a variable in the patient outcome dataset for when the patient outcome occurred? Is this “time” variable a time stamp when the patient outcome was entered into the system, by date and shift, or by date-only?
  - What is the name of the system(s) you use to capture these data?
  - How are adverse events impacted by staffing?
  - Who would we talk to in order to get this information?
